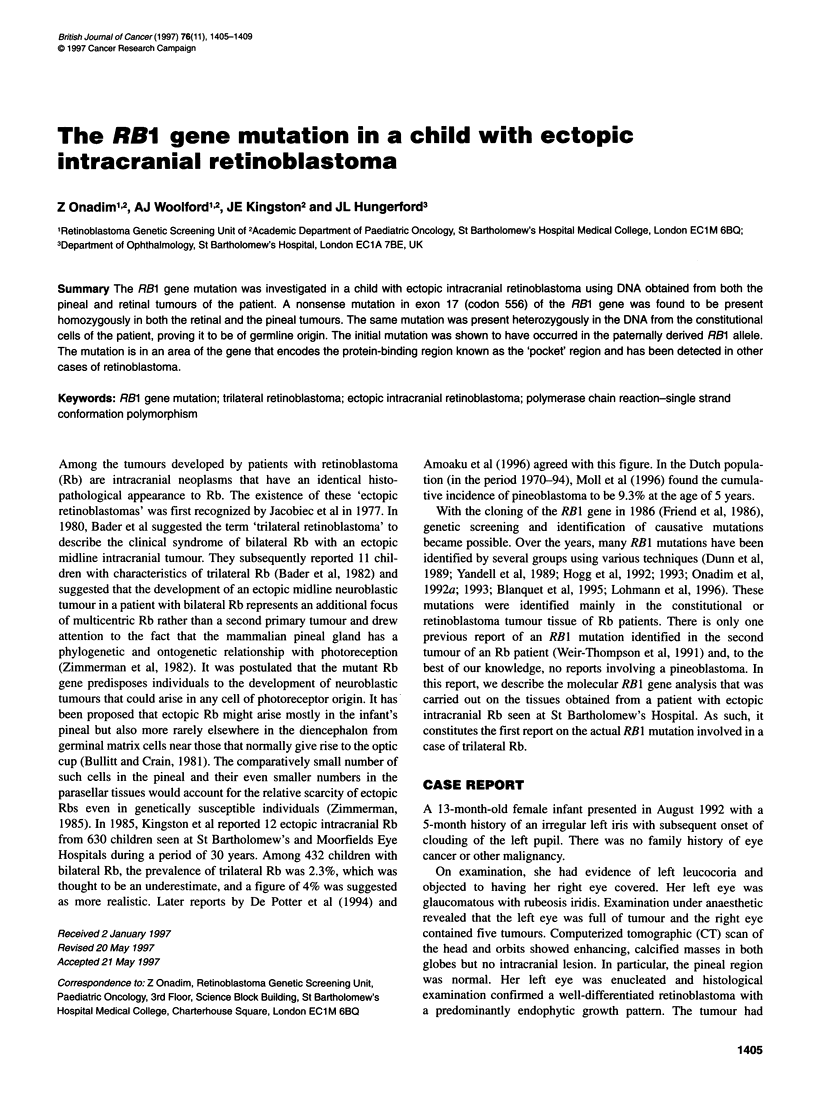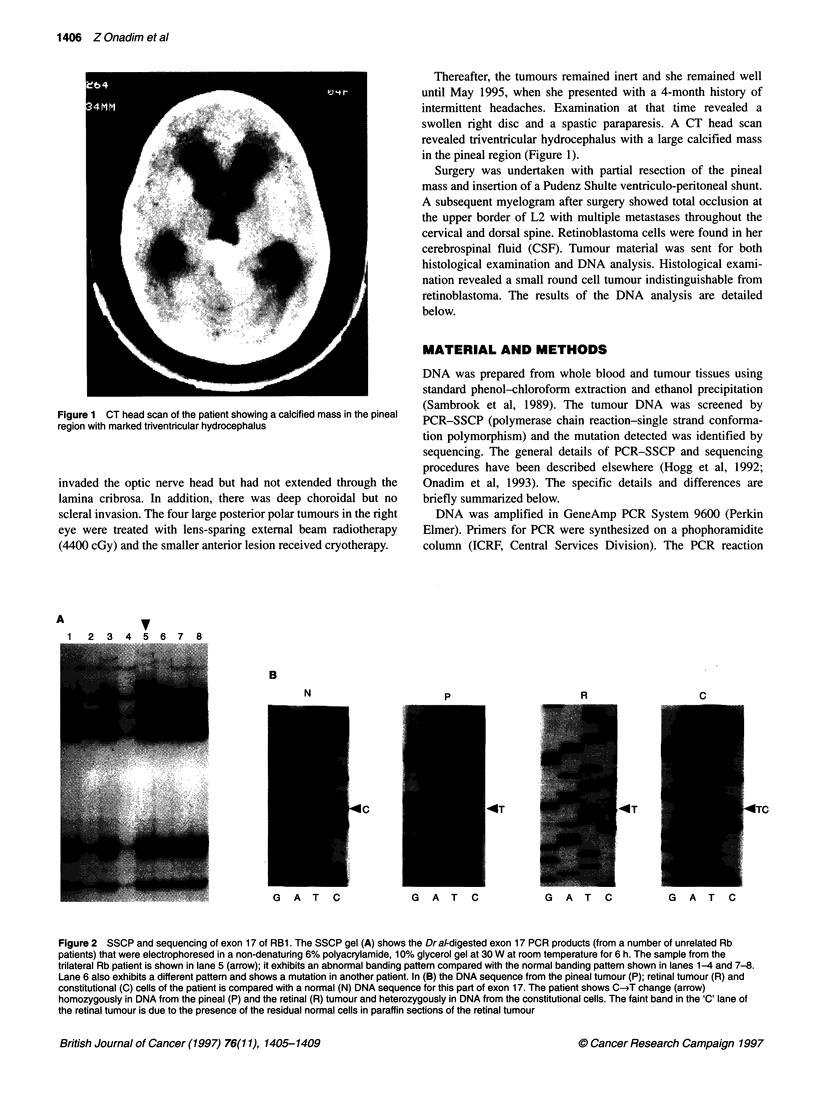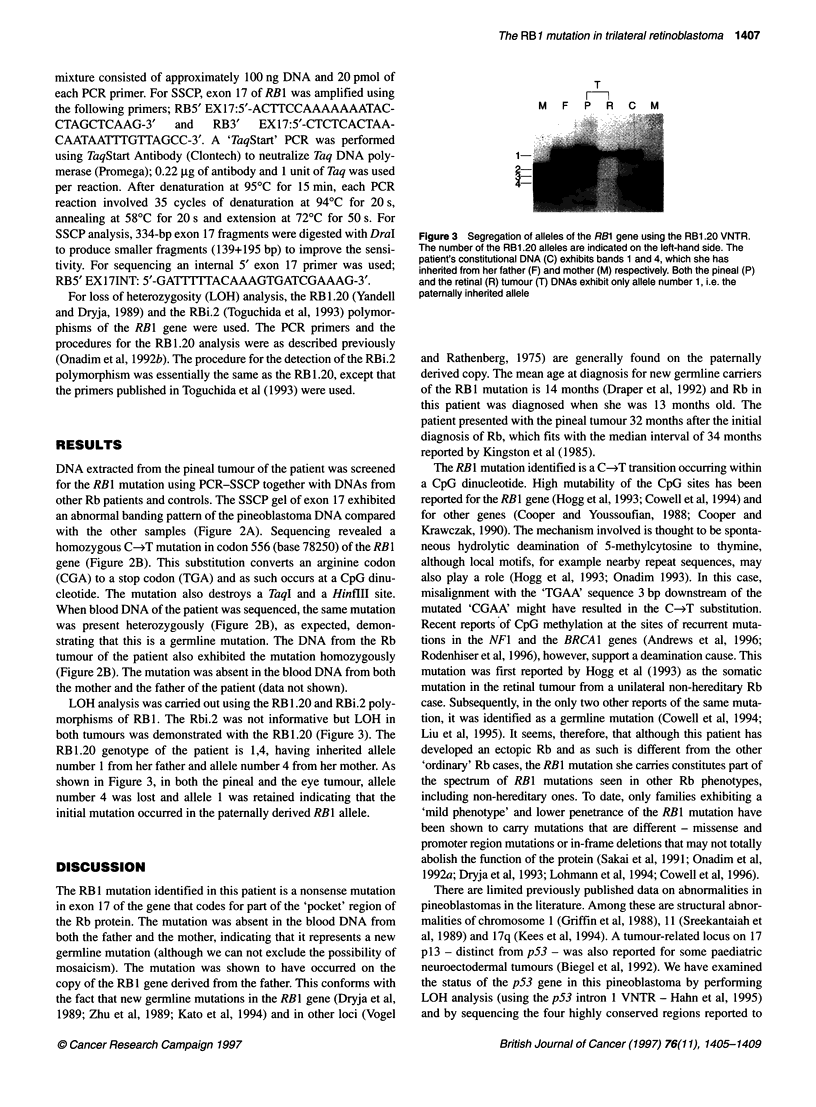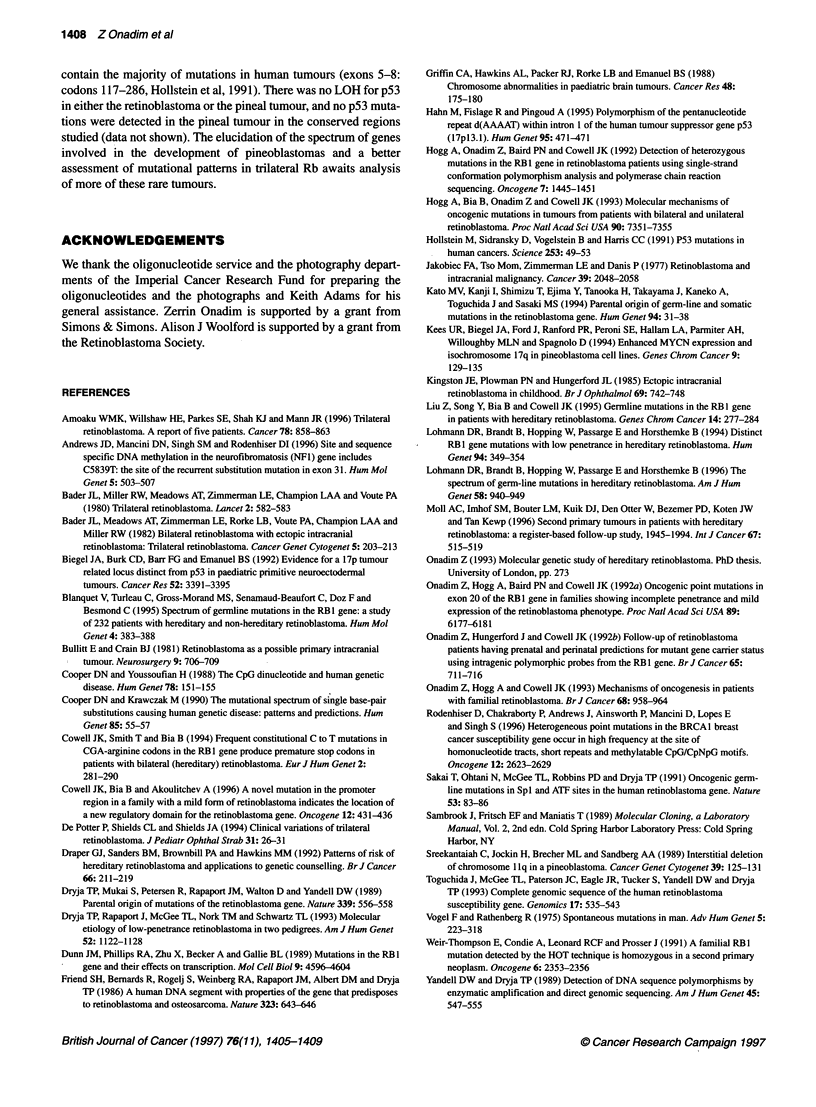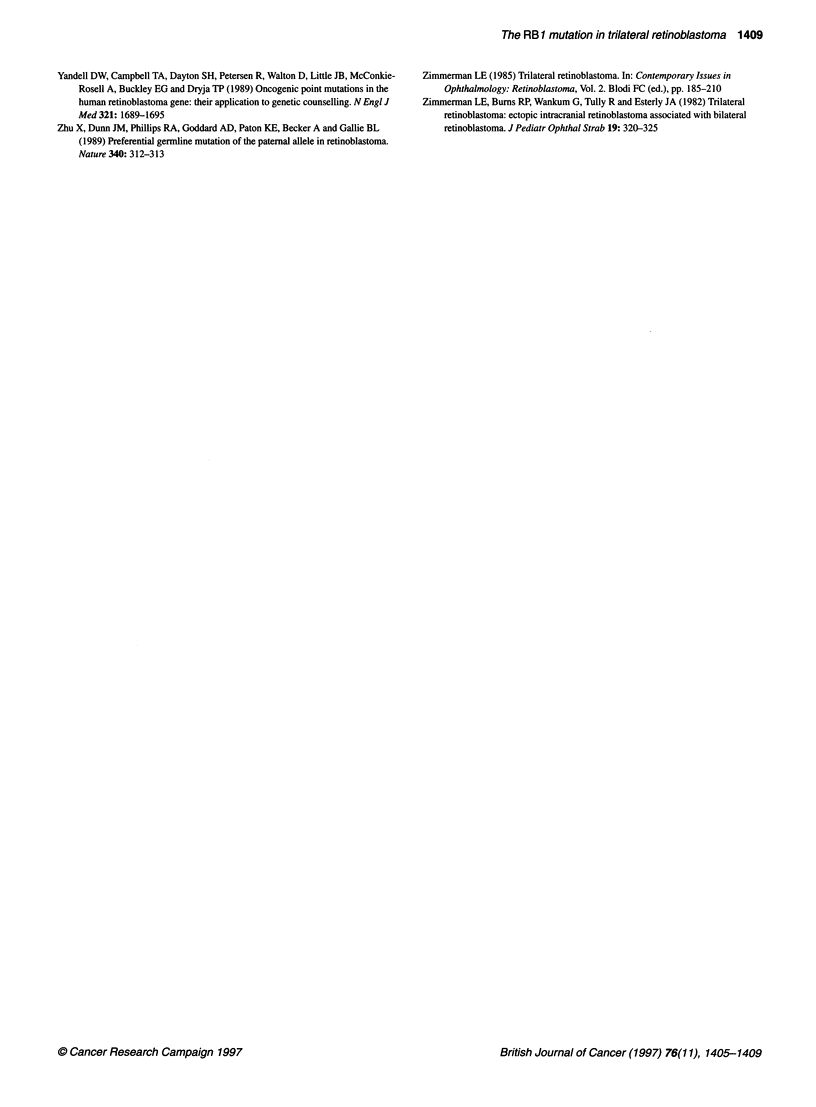# The RB1 gene mutation in a child with ectopic intracranial retinoblastoma.

**DOI:** 10.1038/bjc.1997.570

**Published:** 1997

**Authors:** Z. Onadim, A. J. Woolford, J. E. Kingston, J. L. Hungerford

**Affiliations:** Academic Department of Paediatric Oncology, St Bartholomew's Hospital Medical College, London, UK.

## Abstract

**Images:**


British Journal of Cancer (1997) 76(11), 1405–1409
© 1997 Cancer Research Campaign

### The *RB*1 gene mutation in a child with ectopic intracranial retinoblastoma

**Z Onadim[1,2], AJ Woolford[1,2], JE Kingston[2] and JL Hungerford[3]**

[1]Retinoblastoma Genetic Screening Unit of [2]Academic Department of Paediatric Oncology, St Bartholomew's Hospital Medical College, London EC1M 6BQ; [3]Department of Ophthalmology, St Bartholomew's Hospital, London EC1A 7BE, UK

**Summary** The *RB*1 gene mutation was investigated in a child with ectopic intracranial retinoblastoma using DNA obtained from both the pineal and retinal tumours of the patient. A nonsense mutation in exon 17 (codon 556) of the *RB*1 gene was found to be present homozygously in both the retinal and the pineal tumours. The same mutation was present heterozygously in the DNA from the constitutional cells of the patient, proving it to be of germline origin. The initial mutation was shown to have occurred in the paternally derived *RB*1 allele. The mutation is in an area of the gene that encodes the protein-binding region known as the 'pocket' region and has been detected in other cases of retinoblastoma.

**Keywords:** *RB*1 gene mutation; trilateral retinoblastoma; ectopic intracranial retinoblastoma; polymerase chain reaction–single strand conformation polymorphism

Among the tumours developed by patients with retinoblastoma (Rb) are intracranial neoplasms that have an identical histopathological appearance to Rb. The existence of these 'ectopic retinoblastomas' was first recognized by Jacobiec et al in 1977. In 1980, Bader et al suggested the term 'trilateral retinoblastoma' to describe the clinical syndrome of bilateral Rb with an ectopic midline intracranial tumour. They subsequently reported 11 children with characteristics of trilateral Rb (Bader et al, 1982) and suggested that the development of an ectopic midline neuroblastic tumour in a patient with bilateral Rb represents an additional focus of multicentric Rb rather than a second primary tumour and drew attention to the fact that the mammalian pineal gland has a phylogenetic and ontogenetic relationship with photoreception (Zimmerman et al, 1982). It was postulated that the mutant Rb gene predisposes individuals to the development of neuroblastic tumours that could arise in any cell of photoreceptor origin. It has been proposed that ectopic Rb might arise mostly in the infant's pineal but also more rarely elsewhere in the diencephalon from germinal matrix cells near those that normally give rise to the optic cup (Bullitt and Crain, 1981). The comparatively small number of such cells in the pineal and their even smaller numbers in the parasellar tissues would account for the relative scarcity of ectopic Rbs even in genetically susceptible individuals (Zimmerman, 1985). In 1985, Kingston et al reported 12 ectopic intracranial Rb from 630 children seen at St Bartholomew's and Moorfields Eye Hospitals during a period of 30 years. Among 432 children with bilateral Rb, the prevalence of trilateral Rb was 2.3%, which was thought to be an underestimate, and a figure of 4% was suggested as more realistic. Later reports by De Potter et al (1994) and Amoaku et al (1996) agreed with this figure. In the Dutch population (in the period 1970–94), Moll et al (1996) found the cumulative incidence of pineoblastoma to be 9.3% at the age of 5 years.

*Received 2 January 1997*
*Revised 20 May 1997*
*Accepted 21 May 1997*

*Correspondence to:* Z Onadim, Retinoblastoma Genetic Screening Unit, Paediatric Oncology, 3rd Floor, Science Block Building, St Bartholomew's Hospital Medical College, Charterhouse Square, London EC1M 6BQ

With the cloning of the *RB*1 gene in 1986 (Friend et al, 1986), genetic screening and identification of causative mutations became possible. Over the years, many *RB*1 mutations have been identified by several groups using various techniques (Dunn et al, 1989; Yandell et al, 1989; Hogg et al, 1992; 1993; Onadim et al, 1992*a*; 1993; Blanquet et al, 1995; Lohmann et al, 1996). These mutations were identified mainly in the constitutional or retinoblastoma tumour tissue of Rb patients. There is only one previous report of an *RB*1 mutation identified in the second tumour of an Rb patient (Weir-Thompson et al, 1991) and, to the best of our knowledge, no reports involving a pineoblastoma. In this report, we describe the molecular *RB*1 gene analysis that was carried out on the tissues obtained from a patient with ectopic intracranial Rb seen at St Bartholomew's Hospital. As such, it constitutes the first report on the actual *RB*1 mutation involved in a case of trilateral Rb.

### CASE REPORT

A 13-month-old female infant presented in August 1992 with a 5-month history of an irregular left iris with subsequent onset of clouding of the left pupil. There was no family history of eye cancer or other malignancy.

On examination, she had evidence of left leucocoria and objected to having her right eye covered. Her left eye was glaucomatous with rubeosis iridis. Examination under anaesthetic revealed that the left eye was full of tumour and the right eye contained five tumours. Computerized tomographic (CT) scan of the head and orbits showed enhancing, calcified masses in both globes but no intracranial lesion. In particular, the pineal region was normal. Her left eye was enucleated and histological examination confirmed a well-differentiated retinoblastoma with a predominantly endophytic growth pattern. The tumour had

**Figure 1** CT head scan of the patient showing a calcified mass in the pineal region with marked triventricular hydrocephalus invaded the optic nerve head but had not extended through the lamina cribrosa. In addition, there was deep choroidal but no scleral invasion. The four large posterior polar tumours in the right eye were treated with lens-sparing external beam radiotherapy (4400 cGy) and the smaller anterior lesion received cryotherapy.

Thereafter, the tumours remained inert and she remained well until May 1995, when she presented with a 4-month history of intermittent headaches. Examination at that time revealed a swollen right disc and a spastic paraparesis. A CT head scan revealed triventricular hydrocephalus with a large calcified mass in the pineal region (Figure 1).

Surgery was undertaken with partial resection of the pineal mass and insertion of a Pudenz Shulte ventriculo-peritoneal shunt. A subsequent myelogram after surgery showed total occlusion at the upper border of L2 with multiple metastases throughout the cervical and dorsal spine. Retinoblastoma cells were found in her cerebrospinal fluid (CSF). Tumour material was sent for both histological examination and DNA analysis. Histological examination revealed a small round cell tumour indistinguishable from retinoblastoma. The results of the DNA analysis are detailed below.

### MATERIAL AND METHODS

DNA was prepared from whole blood and tumour tissues using standard phenol–chloroform extraction and ethanol precipitation (Sambrook et al, 1989). The tumour DNA was screened by PCR–SSCP (polymerase chain reaction–single strand conformation polymorphism) and the mutation detected was identified by sequencing. The general details of PCR–SSCP and sequencing procedures have been described elsewhere (Hogg et al, 1992; Onadim et al, 1993). The specific details and differences are briefly summarized below.

DNA was amplified in GeneAmp PCR System 9600 (Perkin Elmer). Primers for PCR were synthesized on a phophoramidite column (ICRF, Central Services Division). The PCR reaction

**Figure 2** SSCP and sequencing of exon 17 of RB1. The SSCP gel (**A**) shows the *Dr aI*-digested exon 17 PCR products (from a number of unrelated Rb patients) that were electrophoresed in a non-denaturing 6% polyacrylamide, 10% glycerol gel at 30 W at room temperature for 6 h. The sample from the trilateral Rb patient is shown in lane 5 (arrow); it exhibits an abnormal banding pattern compared with the normal banding pattern shown in lanes 1–4 and 7–8. Lane 6 also exhibits a different pattern and shows a mutation in another patient. In (**B**) the DNA sequence from the pineal tumour (P); retinal tumour (R) and constitutional (C) cells of the patient is compared with a normal (N) DNA sequence for this part of exon 17. The patient shows C→T change (arrow) homozygously in DNA from the pineal (P) and the retinal (R) tumour and heterozygously in DNA from the constitutional cells. The faint band in the 'C' lane of the retinal tumour is due to the presence of the residual normal cells in paraffin sections of the retinal tumour

© Cancer Research Campaign 1997

mixture consisted of approximately 100 ng DNA and 20 pmol of each PCR primer. For SSCP, exon 17 of *RB*1 was amplified using the following primers; RB5′ EX17:5′-ACTTCCAAAAAAATAC-CTAGCTCAAG-3′ and RB3′ EX17:5′-CTCTCACTAA-CAATAATTTGTTAGCC-3′. A '*Taq*Start' PCR was performed using *Taq*Start Antibody (Clontech) to neutralize *Taq* DNA polymerase (Promega); 0.22 μg of antibody and 1 unit of *Taq* was used per reaction. After denaturation at 95°C for 15 min, each PCR reaction involved 35 cycles of denaturation at 94°C for 20 s, annealing at 58°C for 20 s and extension at 72°C for 50 s. For SSCP analysis, 334-bp exon 17 fragments were digested with *Dra*I to produce smaller fragments (139+195 bp) to improve the sensitivity. For sequencing an internal 5′ exon 17 primer was used; RB5′ EX17INT: 5′-GATTTTTACAAAGTGATCGAAAG-3′.

For loss of heterozygosity (LOH) analysis, the RB1.20 (Yandell and Dryja, 1989) and the RBi.2 (Toguchida et al, 1993) polymorphisms of the *RB*1 gene were used. The PCR primers and the procedures for the RB1.20 analysis were as described previously (Onadim et al, 1992*b*). The procedure for the detection of the RBi.2 polymorphism was essentially the same as the RB1.20, except that the primers published in Toguchida et al (1993) were used.

### RESULTS

DNA extracted from the pineal tumour of the patient was screened for the *RB*1 mutation using PCR–SSCP together with DNAs from other Rb patients and controls. The SSCP gel of exon 17 exhibited an abnormal banding pattern of the pineoblastoma DNA compared with the other samples (Figure 2A). Sequencing revealed a homozygous C→T mutation in codon 556 (base 78250) of the *RB*1 gene (Figure 2B). This substitution converts an arginine codon (CGA) to a stop codon (TGA) and as such occurs at a CpG dinucleotide. The mutation also destroys a *Taq*I and a *Hinf*III site. When blood DNA of the patient was sequenced, the same mutation was present heterozygously (Figure 2B), as expected, demonstrating that this is a germline mutation. The DNA from the Rb tumour of the patient also exhibited the mutation homozygously (Figure 2B). The mutation was absent in the blood DNA from both the mother and the father of the patient (data not shown).

LOH analysis was carried out using the RB1.20 and RBi.2 polymorphisms of RB1. The Rbi.2 was not informative but LOH in both tumours was demonstrated with the RB1.20 (Figure 3). The RB1.20 genotype of the patient is 1,4, having inherited allele number 1 from her father and allele number 4 from her mother. As shown in Figure 3, in both the pineal and the eye tumour, allele number 4 was lost and allele 1 was retained indicating that the initial mutation occurred in the paternally derived *RB*1 allele.

### DISCUSSION

The RB1 mutation identified in this patient is a nonsense mutation in exon 17 of the gene that codes for part of the 'pocket' region of the Rb protein. The mutation was absent in the blood DNA from both the father and the mother, indicating that it represents a new germline mutation (although we can not exclude the possibility of mosaicism). The mutation was shown to have occurred on the copy of the RB1 gene derived from the father. This conforms with the fact that new germline mutations in the *RB*1 gene (Dryja et al, 1989; Zhu et al, 1989; Kato et al, 1994) and in other loci (Vogel

**Figure 3** Segregation of alleles of the *RB*1 gene using the RB1.20 VNTR. The number of the RB1.20 alleles are indicated on the left-hand side. The patient's constitutional DNA (C) exhibits bands 1 and 4, which she has inherited from her father (F) and mother (M) respectively. Both the pineal (P) and the retinal (R) tumour (T) DNAs exhibit only allele number 1, i.e. the paternally inherited allele and Rathenberg, 1975) are generally found on the paternally derived copy. The mean age at diagnosis for new germline carriers of the RB1 mutation is 14 months (Draper et al, 1992) and Rb in this patient was diagnosed when she was 13 months old. The patient presented with the pineal tumour 32 months after the initial diagnosis of Rb, which fits with the median interval of 34 months reported by Kingston et al (1985).

The *RB*1 mutation identified is a C→T transition occurring within a CpG dinucleotide. High mutability of the CpG sites has been reported for the *RB*1 gene (Hogg et al, 1993; Cowell et al, 1994) and for other genes (Cooper and Youssoufian, 1988; Cooper and Krawczak, 1990). The mechanism involved is thought to be spontaneous hydrolytic deamination of 5-methylcytosine to thymine, although local motifs, for example nearby repeat sequences, may also play a role (Hogg et al, 1993; Onadim 1993). In this case, misalignment with the 'TGAA' sequence 3 bp downstream of the mutated 'CGAA' might have resulted in the C→T substitution. Recent reports of CpG methylation at the sites of recurrent mutations in the *NF*1 and the *BRCA*1 genes (Andrews et al, 1996; Rodenhiser et al, 1996), however, support a deamination cause. This mutation was first reported by Hogg et al (1993) as the somatic mutation in the retinal tumour from a unilateral non-hereditary Rb case. Subsequently, in the only two other reports of the same mutation, it was identified as a germline mutation (Cowell et al, 1994; Liu et al, 1995). It seems, therefore, that although this patient has developed an ectopic Rb and as such is different from the other 'ordinary' Rb cases, the *RB*1 mutation she carries constitutes part of the spectrum of *RB*1 mutations seen in other Rb phenotypes, including non-hereditary ones. To date, only families exhibiting a 'mild phenotype' and lower penetrance of the *RB*1 mutation have been shown to carry mutations that are different – missense and promoter region mutations or in-frame deletions that may not totally abolish the function of the protein (Sakai et al, 1991; Onadim et al, 1992*a*; Dryja et al, 1993; Lohmann et al, 1994; Cowell et al, 1996).

There are limited previously published data on abnormalities in pineoblastomas in the literature. Among these are structural abnormalities of chromosome 1 (Griffin et al, 1988), 11 (Sreekantaiah et al, 1989) and 17q (Kees et al, 1994). A tumour-related locus on 17 p13 – distinct from *p53* – was also reported for some paediatric neuroectodermal tumours (Biegel et al, 1992). We have examined the status of the *p53* gene in this pineoblastoma by performing LOH analysis (using the *p53* intron 1 VNTR – Hahn et al, 1995) and by sequencing the four highly conserved regions reported to

© Cancer Research Campaign 1997

contain the majority of mutations in human tumours (exons 5–8: codons 117–286, Hollstein et al, 1991). There was no LOH for p53 in either the retinoblastoma or the pineal tumour, and no p53 mutations were detected in the pineal tumour in the conserved regions studied (data not shown). The elucidation of the spectrum of genes involved in the development of pineoblastomas and a better assessment of mutational patterns in trilateral Rb awaits analysis of more of these rare tumours.

### ACKNOWLEDGEMENTS

We thank the oligonucleotide service and the photography departments of the Imperial Cancer Research Fund for preparing the oligonucleotides and the photographs and Keith Adams for his general assistance. Zerrin Onadim is supported by a grant from Simons & Simons. Alison J Woolford is supported by a grant from the Retinoblastoma Society.

### REFERENCES

Amoaku WMK, Willshaw HE, Parkes SE, Shah KJ and Mann JR (1996) Trilateral retinoblastoma. A report of five patients. *Cancer* **78:** 858–863

Andrews JD, Mancini DN, Singh SM and Rodenhiser DI (1996) Site and sequence specific DNA methylation in the neurofibromatosis (NF1) gene includes C5839T: the site of the recurrent substitution mutation in exon 31. *Hum Mol Genet* **5:** 503–507

Bader JL, Miller RW, Meadows AT, Zimmerman LE, Champion LAA and Voute PA (1980) Trilateral retinoblastoma. *Lancet* **2:** 582–583

Bader JL, Meadows AT, Zimmerman LE, Rorke LB, Voute PA, Champion LAA and Miller RW (1982) Bilateral retinoblastoma with ectopic intracranial retinoblastoma: Trilateral retinoblastoma. *Cancer Genet Cytogenet* **5:** 203–213

Biegel JA, Burk CD, Barr FG and Emanuel BS (1992) Evidence for a 17p tumour related locus distinct from p53 in paediatric primitive neuroectodermal tumours. *Cancer Res* **52:** 3391–3395

Blanquet V, Turleau C, Gross-Morand MS, Senamaud-Beaufort C, Doz F and Besmond C (1995) Spectrum of germline mutations in the RB1 gene: a study of 232 patients with hereditary and non-hereditary retinoblastoma. *Hum Mol Genet* **4:** 383–388

Bullitt E and Crain BJ (1981) Retinoblastoma as a possible primary intracranial tumour. *Neurosurgery* **9:** 706–709

Cooper DN and Youssoufian H (1988) The CpG dinucleotide and human genetic disease. *Hum Genet* **78:** 151–155

Cooper DN and Krawczak M (1990) The mutational spectrum of single base-pair substitutions causing human genetic disease: patterns and predictions. *Hum Genet* **85:** 55–57

Cowell JK, Smith T and Bia B (1994) Frequent constitutional C to T mutations in CGA-arginine codons in the RB1 gene produce premature stop codons in patients with bilateral (hereditary) retinoblastoma. *Eur J Hum Genet* **2:** 281–290

Cowell JK, Bia B and Akoulitchev A (1996) A novel mutation in the promoter region in a family with a mild form of retinoblastoma indicates the location of a new regulatory domain for the retinoblastoma gene. *Oncogene* **12:** 431–436

De Potter P, Shields CL and Shields JA (1994) Clinical variations of trilateral retinoblastoma. *J Pediatr Ophthal Strab* **31:** 26–31

Draper GJ, Sanders BM, Brownbill PA and Hawkins MM (1992) Patterns of risk of hereditary retinoblastoma and applications to genetic counselling. *Br J Cancer* **66:** 211–219

Dryja TP, Mukai S, Petersen R, Rapaport JM, Walton D and Yandell DW (1989) Parental origin of mutations of the retinoblastoma gene. *Nature* **339:** 556–558

Dryja TP, Rapaport J, McGee TL, Nork TM and Schwartz TL (1993) Molecular etiology of low-penetrance retinoblastoma in two pedigrees. *Am J Hum Genet* **52:** 1122–1128

Dunn JM, Phillips RA, Zhu X, Becker A and Gallie BL (1989) Mutations in the RB1 gene and their effects on transcription. *Mol Cell Biol* **9:** 4596–4604

Friend SH, Bernards R, Rogelj S, Weinberg RA, Rapaport JM, Albert DM and Dryja TP (1986) A human DNA segment with properties of the gene that predisposes to retinoblastoma and osteosarcoma. *Nature* **323:** 643–646

Griffin CA, Hawkins AL, Packer RJ, Rorke LB and Emanuel BS (1988) Chromosome abnormalities in paediatric brain tumours. *Cancer Res* **48:** 175–180

Hahn M, Fislage R and Pingoud A (1995) Polymorphism of the pentanucleotide repeat d(AAAAT) within intron 1 of the human tumour suppressor gene p53 (17p13.1). *Hum Genet* **95:** 471–471

Hogg A, Onadim Z, Baird PN and Cowell JK (1992) Detection of heterozygous mutations in the RB1 gene in retinoblastoma patients using single-strand conformation polymorphism analysis and polymerase chain reaction sequencing. *Oncogene* **7:** 1445–1451

Hogg A, Bia B, Onadim Z and Cowell JK (1993) Molecular mechanisms of oncogenic mutations in tumours from patients with bilateral and unilateral retinoblastoma. *Proc Natl Acad Sci USA* **90:** 7351–7355

Hollstein M, Sidransky D, Vogelstein B and Harris CC (1991) P53 mutations in human cancers. *Science* **253:** 49–53

Jakobiec FA, Tso Mom, Zimmerman LE and Danis P (1977) Retinoblastoma and intracranial malignancy. *Cancer* **39:** 2048–2058

Kato MV, Kanji I, Shimizu T, Ejima Y, Tanooka H, Takayama J, Kaneko A, Toguchida J and Sasaki MS (1994) Parental origin of germ-line and somatic mutations in the retinoblastoma gene. *Hum Genet* **94:** 31–38

Kees UR, Biegel JA, Ford J, Ranford PR, Peroni SE, Hallam LA, Parmiter AH, Willoughby MLN and Spagnolo D (1994) Enhanced MYCN expression and isochromosome 17q in pineoblastoma cell lines. *Genes Chrom Cancer* **9:** 129–135

Kingston JE, Plowman PN and Hungerford JL (1985) Ectopic intracranial retinoblastoma in childhood. *Br J Ophthalmol* **69:** 742–748

Liu Z, Song Y, Bia B and Cowell JK (1995) Germline mutations in the RB1 gene in patients with hereditary retinoblastoma. *Genes Chrom Cancer* **14:** 277–284

Lohmann DR, Brandt B, Hopping W, Passarge E and Horsthemke B (1994) Distinct RB1 gene mutations with low penetrance in hereditary retinoblastoma. *Hum Genet* **94:** 349–354

Lohmann DR, Brandt B, Hopping W, Passarge E and Horsthemke B (1996) The spectrum of germ-line mutations in hereditary retinoblastoma. *Am J Hum Genet* **58:** 940–949

Moll AC, Imhof SM, Bouter LM, Kuik DJ, Den Otter W, Bezemer PD, Koten JW and Tan Kewp (1996) Second primary tumours in patients with hereditary retinoblastoma: a register-based follow-up study, 1945–1994. *Int J Cancer* **67:** 515–519

Onadim Z (1993) Molecular genetic study of hereditary retinoblastoma. PhD thesis. University of London, pp. 273

Onadim Z, Hogg A, Baird PN and Cowell JK (1992*a*) Oncogenic point mutations in exon 20 of the RB1 gene in families showing incomplete penetrance and mild expression of the retinoblastoma phenotype. *Proc Natl Acad Sci USA* **89:** 6177–6181

Onadim Z, Hungerford J and Cowell JK (1992*b*) Follow-up of retinoblastoma patients having prenatal and perinatal predictions for mutant gene carrier status using intragenic polymorphic probes from the RB1 gene. *Br J Cancer* **65:** 711–716

Onadim Z, Hogg A and Cowell JK (1993) Mechanisms of oncogenesis in patients with familial retinoblastoma. *Br J Cancer* **68:** 958–964

Rodenhiser D, Chakraborty P, Andrews J, Ainsworth P, Mancini D, Lopes E and Singh S (1996) Heterogeneous point mutations in the BRCA1 breast cancer susceptibility gene occur in high frequency at the site of homonucleotide tracts, short repeats and methylatable CpG/CpNpG motifs. *Oncogene* **12:** 2623–2629

Sakai T, Ohtani N, McGee TL, Robbins PD and Dryja TP (1991) Oncogenic germ-line mutations in Sp1 and ATF sites in the human retinoblastoma gene. *Nature* **53:** 83–86

Sambrook J, Fritsch EF and Maniatis T (1989) *Molecular Cloning, a Laboratory Manual*, Vol. 2, 2nd edn. Cold Spring Harbor Laboratory Press: Cold Spring Harbor, NY

Sreekantaiah C, Jockin H, Brecher ML and Sandberg AA (1989) Interstitial deletion of chromosome 11q in a pineoblastoma. *Cancer Genet Cytogenet* **39:** 125–131

Toguchida J, McGee TL, Paterson JC, Eagle JR, Tucker S, Yandell DW and Dryja TP (1993) Complete genomic sequence of the human retinoblastoma susceptibility gene. *Genomics* **17:** 535–543

Vogel F and Rathenberg R (1975) Spontaneous mutations in man. *Adv Hum Genet* **5:** 223–318

Weir-Thompson E, Condie A, Leonard RCF and Prosser J (1991) A familial RB1 mutation detected by the HOT technique is homozygous in a second primary neoplasm. *Oncogene* **6:** 2353–2356

Yandell DW and Dryja TP (1989) Detection of DNA sequence polymorphisms by enzymatic amplification and direct genomic sequencing. *Am J Hum Genet* **45:** 547–555

© Cancer Research Campaign 1997

Yandell DW, Campbell TA, Dayton SH, Petersen R, Walton D, Little JB, McConkie-Rosell A, Buckley EG and Dryja TP (1989) Oncogenic point mutations in the human retinoblastoma gene: their application to genetic counselling. *N Engl J Med* **321:** 1689–1695

Zhu X, Dunn JM, Phillips RA, Goddard AD, Paton KE, Becker A and Gallie BL (1989) Preferential germline mutation of the paternal allele in retinoblastoma. *Nature* **340:** 312–313

Zimmerman LE (1985) Trilateral retinoblastoma. In: *Contemporary Issues in Ophthalmology: Retinoblastoma*, Vol. 2. Blodi FC (ed.), pp. 185–210

Zimmerman LE, Burns RP, Wankum G, Tully R and Esterly JA (1982) Trilateral retinoblastoma: ectopic intracranial retinoblastoma associated with bilateral retinoblastoma. *J Pediatr Ophthal Strab* **19:** 320–325

© Cancer Research Campaign 1997